# Could the Adriatic islands of Croatia be heading in the same direction as the Canary Islands?

**DOI:** 10.3325/cmj.2019.60.58

**Published:** 2019-02

**Authors:** Jan Brož, Samuel Ortega Sarmiento, Dario Rahelić

**Affiliations:** 1 Department of Internal Medicine, Second Faculty of Medicine, Charles University, Prague, the Czech Republic *zorb@seznam.cz*; 2 Institute for Applied Microelectronics, University of Las Palmas de Gran Canaria, Las Palmas, Spain; 3 Department of Endocrinology, Diabetes and Clinical Pharmacology, Dubrava University Hospital, Zagreb, Croatia

To the Editor: We read with interest Rehberg and colleagues’ article “Mortality Patterns in the Southern Adriatic Islands of Croatia: a Registry-based Study,” recently published in the *Croatian Medical Journal* (1). The authors compared the mortality patterns between the Southern Adriatic islands of Croatia and two predominantly coastal Croatian mainland counties. One of the major findings was that although the island inhabitants currently had a longer lifespan than the mainland population, there was no difference in life expectancy at birth. The leading cause of death in the islands was cardiovascular disease, with higher percentage rates in men and lower in women compared with the mainland. The authors attributed the increasingly unfavorable mortality patterns in the recent generations of islanders to the diminishing adherence to the Mediterranean diet and lifestyle.

Although we have no critical remarks regarding the study itself, we would like to make a suggestion for the authors. Marcelino-Rodríguez et al (2) found that the diabetes prevalence in the Canary Islands was higher than in ten other Spanish regions in age groups younger than 64, while it was similar in older age groups. Since 1981, the Canary Islands had the highest diabetes-related mortality in Spain, which peaked in 2011 in both sexes. Diabetes-related mortality in nine of other ten Spanish regions decreased from 1981 to 2011. The only group that did not consistently follow this trend were male Canary Islanders.

Although we are aware of the differences between the Southern Adriatic islands of Croatia and the Canary Islands, both these regions have highly developed tourist industries, which may in the long-term influence the lifestyle in the area and increase diabetes prevalence with all its consequences. As diabetes prevalence trends are not available, we respectfully suggest the authors to consider analyzing diabetes-related mortality in their data set, as this could reveal interesting results related to this disease and indirectly suggest its possible connection with increased cardiovascular mortality.
